# Biological Research marks its 50th year by joining BioMed Central

**DOI:** 10.1186/0717-6287-47-1

**Published:** 2014-03-26

**Authors:** Manuel J Santos

**Affiliations:** Departments of Cell and Molecular Biology and Pediatrics, Faculties of Biological Sciences and Medicine, Pontificia Universidad Catolica de Chile, Casilla, 114-D Santiago Chile

## Editorial

*Biological Research’s* celebrates its 50th year in 2014. The Chilean Biology Society and I are delighted to mark this milestone by entering a new era in which the journal is published by BioMed Central. The journal has a long history publishing research across the broad spectrum of experimental biology, having been founded in 1964 as “*Archivos de Biología y Medicina Experimentales*” and renamed in 1992 to its current title.

We are certain that, by being associated with this prestigious publishing house, *Biological Research* will have greater visibility, which will attract new authors from around the world to publish in the journal and gather new readers. The resultant increase in downloads and impact should lead to a rise in citations and growth of the Impact Factor, currently 1.13. The journal is indexed in PubMed and Scopus, and published articles are archived in PubMed Central, SciELO and other accessible full text repositories. This shows that the journal is one of the scientific publications of Latin American-origin with greatest international impact.

We welcome original articles and invited reviews in diverse fields of experimental biology, including biochemistry, bioinformatics, biotechnology, cell biology, cancer, chemical biology, developmental biology, evolutionary biology, genetics, genomics, immunology, marine biology, microbiology, molecular biology, neurosciences, plant biology, physiology, stem cells, structural biology and systems biology.

In an attempt to continually improve the impact and competitiveness of this journal, manuscripts will receive immediate attention upon arrival. Submitted manuscripts will be handled by an Associate Editor and evaluated by at least two independent reviewers, who are either members of the Editorial Board or *ad hoc* referees. The reviewers, experts in the area to which the manuscript pertains, will critically analyse the manuscript in a timely manner. Acceptance for publication is based on the scientific content, originality and presentation of the material. Accepted manuscripts will be published electronically within a few weeks of their final approval date.

We will continue to publish some special issues in *Biological Research,* dedicated to a specific area. In the recent past we published two special issues focused on ‘Stem Cell Research’ (Biol Res 45(3), 2012) [1] and on ‘Biotechnology’ (Biol Res 46(4), 2013) [2] respectively, which have been highly cited. In 2014, we will publish a special issue on ‘Neuroscience Research’.

Among the first articles in volume 47 of *Biological Research,* published by BioMed Central as open access manuscripts, we include a variety of papers covering different areas of experimental biology. They include manuscripts on biomedicine (such as human cancer genetics (Ramalho et al., Brazil) [3]; genetic polymorphisms associated with human nutrition, (Fang et al., China) [4]; animal physiology (oxidative stress and nutrition, Otunola et al., South Africa) [5]; molecular biology (new molecular biotechnology tools to attack spider toxins, Sapag et al., Chile) [6]; plant biology (antinflammatory effect of a native medicinal plant; Morales et al., Chile) [7]; and animal experimentation (cardiac function in rats, Zheng et al., China) [8].

Technological advances support both editing and raising the visibility of journals. We already use Twitter ( http://www.twitter.com/Bio_Research) and Facebook ( http://www.facebook.com/Biological.ResearchCL) to aid the dissemination of the scope and content of *Biological Research*, as well as the Chilean Biology Society newsletter. Now the journal is published by BioMed Central, we welcome comments directly on an article, to which an author may wish to reply. Article metrics will be provided for all articles to help readers assess the overall importance and impact of an article in terms of citations, downloads, and the discussion and debate about the article taking place on the web.

We expect that this new alliance between the Chilean Biology Society and BioMed Central will strengthen the international competitiveness of *Biological Research* and its contents, and facilitate access to the journal for international researchers, thus increasing the number of articles submitted for review. We welcome your submissions as we enter this new phase in the journal’s history.

Manuel J. Santos, MD, PhD,

Editor of *Biological Research*
